# WHY WE NEED A REPRESENTATIVE ORGANIZATION OF ACADEMIC PRM IN EUROPE, AND WHY WE NEED IT NOW

**DOI:** 10.2340/jrm.v57.42369

**Published:** 2025-01-03

**Authors:** Gerold STUCKI, Henk J. STAM, Francesca GIMIGLIANO, Stefano NEGRINI

**Affiliations:** 1 Faculty of Health Sciences and Medicine, University of Lucerne, Lucerne, Switzerland; 2 Center for Rehabilitation in Global Health Systems, WHO Collaborating Center, University of Lucerne, Lucerne, Switzerland; 3 Swiss Paraplegic Research, Nottwil, Switzerland; 4 Department of Biomedical, Surgical, and Dental Sciences, University of Milan “La Statale”, Milan, Italy; 5 IRCCS Istituto Ortopedico Galeazzi, Milan, Italy; 6 Department of Rehabilitation Medicine Erasmus MC, University Medical Center, Rotterdam, The Netherlands; 7 Department of Mental and Physical Health and Preventive Medicine, University of Campania “Luigi Vanvitelli”, Naples, Italy

**Keywords:** academic medical centres, education, medical faculty, physical and rehabilitation medicine, professional competence, rehabilitation

## Abstract

The growing relevance of rehabilitation in healthcare to address increasing patient needs necessitates robust Physical and Rehabilitation Medicine (PRM) integration into medical education and practice. Academic PRM, rooted in medical faculties, is vital for developing the medical speciality as an academic discipline across Europe, where it faces challenges, including limited representation in medical schools and competition for resources with established disciplines. This commentary advocates establishing a representative organization for academic PRM in Europe.

Currently, no organization adequately represents academic PRM at the European level. The lack of academic chairs and departments in some countries undermines PRM’s credibility and visibility, making it less attractive to students and prospective academics. An organized effort could provide a platform for knowledge exchange, policy formulation, and advocacy, ultimately strengthening the discipline’s presence in medical faculties.

Immediate action is crucial, particularly in light of the WHO’s call for action 2030 and its 2023 resolution emphasizing the need for rehabilitation within health systems. A representative European academic PRM organization could advocate for education on rehabilitation across all medical specialities and enhance the understanding of functioning as a health indicator. It would also support the development of national academic organizations across Europe and facilitate collaboration with other stakeholders, including patient organizations, rehabilitation professionals, and funding agencies.

Critical considerations for creating this organization include defining core activities, establishing governance principles focused on representativity and inclusion, and fostering relationships with existing national, European, and international organizations of PRM and academic medicine. By leveraging successful models like the Association of Academic Physiatrists in the United States, Europe can develop a robust and credible voice for academic PRM. This initiative is timely and necessary to capitalize on the current momentum and ensure the future of academic PRM in Europe.

## LAY ABSTRACT

Rehabilitation is becoming an essential part of healthcare, and Physical and Rehabilitation Medicine is crucial in this development. Physical and Rehabilitation Medicine helps integrate rehabilitation into medical education and research across Europe, but the field faces challenges in gaining visibility and resources, often competing with more established medical disciplines.

Currently, there is no representative organization for academic Physical and Rehabilitation Medicine in Europe, which makes it harder for the field to grow. A European Physical and Rehabilitation Medicine organization would help by providing support, promoting collaboration, and advocating for the importance of physical and rehabilitation medicine in medical faculties and more broadly rehabilitation in healthcare. This would improve visibility, attract aspiring academics to the field of academic Physical and Rehabilitation Medicine, and secure resources for rehabilitation programs.

The recent WHO resolution on strengthening rehabilitation offers an important opportunity for Physical and Rehabilitation Medicine to contribute to a healthcare system that is responsive to persons' healthcare needs in the future. A representative organization of academic Physical and Rehabilitation Medicine can shape the future rehabilitation in Europe.

Given the increasing importance of rehabilitation as a health strategy, it is essential to ground and strengthen rehabilitation in medicine and in medical education. This can only be achieved by the academic discipline of Physical and Rehabilitation Medicine (PRM) (1, 2).

Academic PRM, anchored in medical faculties, is the foundation for the development of the medical speciality of PRM as an academic discipline in Europe.

From an institutional perspective, academic PRM physicians contribute to all activities of medical faculties as well as national and European educational and research organizations.

From the perspective of academic capacity building, academic PRM physicians are responsible for the scientific education and training of clinician scientists and the training and mentoring in academic leadership of prospective PRM academics. They take responsibility for the 4 pillars of an academic medical discipline: education and training, research, patient care, and academic administration.

From the perspective of education and training, academic PRM physicians are responsible for the medical education in PRM, rehabilitation, and functioning of medical students. They contribute to the training and continuing medical education of PRM specialists by PRM academics.

From the perspective of *research*, academic PRM physicians are responsible for developing the scientific foundation of the medical speciality of PRM, rehabilitation, and functioning.

From the perspective of *patient care*, academic PRM physicians are responsible for the integration of evidence and the exploration and evaluation of innovative care approaches and models.

From the perspective of *academic administration*, academic PRM physicians take responsibility for management of all aspects relevant to their academic discipline in the context of their faculties.

PRM academics share the responsibility to (*i*) ensure the development of the medical speciality of PRM as an academic discipline, (*ii*) establish rehabilitation as a health strategy in medicine, and (*iii*) strengthen rehabilitation in health systems to address the functioning needs of the population. This raises the question of how PRM academics can best collaborate at the national, European, and international levels to achieve these goals.

In this commentary, we first argue why we need a representative organization of academic PRM in Europe. We will then argue why we need to act now. Finally, we will present some initial considerations for the successful development of such an organization in concert with emerging national organizations for academic PRM and the newly founded Academic Forum of the International Society of Physical and Rehabilitation Medicine (ISPRM) at the international level.

## WHY WE NEED A REPRESENTATIVE ORGANIZATION OF ACADEMIC PRM AT THE EUROPEAN LEVEL

In the current European context, there is a need for a representative organization for academic PRM that has the legitimacy to advocate for the sustainable representation and development of academic PRM in all medical faculties. In addition, there is a need for an organization that will lobby for the allocation of appropriate resources for rehabilitation in medical education, research, and academically based clinical care.

Currently, there is no independent and representative academic organization for PRM in Europe that can fulfil this role. The mandate of the UEMS Section and Board is the medical specialty of PRM while the focus of the European Society of PRM (ESPRM) is knowledge translation into clinical practice. The European Academy of Rehabilitation Medicine (EARM) is a non-representative organization of up to 50 physicians nominated by its members and elected by its assembly.

With the striking exception of some countries, the academic discipline of PRM has grown in Europe in recent years. However, some intrinsic characteristics of this discipline have resulted in significant challenges.

First, PRM is one of the youngest academic disciplines and is in competition for resources and time dedicated to curriculums for rehabilitation with long established academic disciplines.

Second, rehabilitation, by its very nature, is relevant to many other academic disciplines and medical specialities. The starting and unique point for academic PRM is an interdisciplinary approach based on the understanding of functioning in the light of health conditions and personal resources as well as interaction with the environment. Instead, the starting point of most other academic disciplines is to approach rehabilitation from a biomedical perspective, which is the pre-eminent approach to medical education and practice in medical faculties. The strength of interdisciplinarity of academic PRM can become a weakness if rehabilitation is claimed by other academic disciplines with a narrow health condition or pathology-oriented perspective.

Third, and arguably the most important challenge, is that academic PRM is not represented across medical faculties in Europe. In some countries, there is no full chair position in PRM and no university department of PRM. This threatens the credibility of PRM as an academic discipline on a par with other academic disciplines. The limited presence of academic PRM in medical faculties is in clear contrast with the presence of the medical speciality of PRM in health systems. As a medical speciality PRM is present in all but one country in the European Union and is one of the 43 most relevant medical specialities represented in the European Union of Medical Specialists (UEMS).

Finally, the competition for talented, passionate, and ambitious students entering an academic discipline is significant. Academic PRM often receives low priority within medical faculties and university hospitals, resulting in a lack of visibility. This reduces its attractiveness for medical students and, as a result, they may not choose to pursue an academic career in PRM. Also, the lack of academic staff and facilities dedicated to PRM hampers the development of talented clinician scientists, leading to a shortage of aspiring PRM academics applying for professorships.

An organization of academic PRM in Europe can address the challenges mentioned above and strengthen academic PRM in several ways.

First, it can serve as a platform for PRM departments and PRM academics in Europe to provide information and facilitate the exchange of knowledge and experience across key areas of academic PRM (see Table I). In light of the important goal to establish academic chairs and departments at every medical faculty in Europe, the members of an organization of academic PRM can provide mutual support and strategies to achieve these goals and strive for the establishment of national organizations for academic PRM (2).

**Table I:** Envisioned activities of a representative academic PRM organization in Europe to be pursued in collaboration with other PRM organizations at the national, European, and international level

Activity	Description
1. PRM, Rehabilitation, and Functioning in Medical Education	Development of curriculum and training programmes in PRM, focusing on integrating rehabilitation and functioning into medical education. Includes both undergraduate and postgraduate training, emphasizing clinical and practical knowledge for future PRM physicians
2. PRM, Rehabilitation, and Functioning in Health Professionals’ Education	Extension of PRM education to other healthcare professionals, such as physical therapists, occupational therapists, nurses, speech and language therapists, psychologists, social workers, etc. Training programmes aim to foster interdisciplinary collaboration and shared knowledge in rehabilitation and functioning
3. Scientific Education and Training of Clinician Scientists in PRM	Establishment of educational pathways and research programmes that train clinician scientists in PRM. Focuses on fostering research skills, critical analysis, and innovation in rehabilitation and functioning
4. Development of Academic Schools and Leadership	Creation and organization of academic schools that offer structured education in areas like evidence-based clinical rehabilitation, human functioning sciences, rehabilitation sciences, and health systems. Includes developing academic leadership programmes to train future leaders in PRM education and research
5. Training and Mentoring in Academic Leadership	Mentoring programmes designed to prepare aspiring PRM academics for leadership roles in education, research, and clinical practice. Emphasizes skill development in academic administration, mentorship, and policy advocacy
6. Scientific Foundations and Cooperative Research Platforms	Developing the scientific foundation of PRM by facilitating cooperative research across national and European platforms. Includes support for multi-centre research projects, grant applications, and collaboration with European-level funding organizations to promote advanced research in rehabilitation
7. Representation in Funding Institutions and Universities	Advocacy for academic PRM representation at the European level in funding institutions and universities. Aims to strengthen the presence of PRM in academic and clinical settings and support research through adequate financial backing
8. Advocacy for Academic PRM Chairs and Departments	Support for the creation and maintenance of PRM departments and academic chairs in universities and medical faculties. Focuses on increasing the visibility and importance of PRM within medical academia, fostering leadership positions in PRM across Europe
9. Training and Continuing Medical Education for PRM Specialists	Contribution of academic PRM physicians to the training and lifelong education of PRM specialists. This includes designing continuing medical education (CME) programmes that keep specialists up to date with the latest practices, research, and innovations in rehabilitation medicine
10. Strengthening Rehabilitation in Health Systems	Strengthening the role of rehabilitation in health systems by focusing on the 6 building blocks of health systems (leadership, service delivery, workforce, financing, information, and technology). Efforts align with the WHO’s resolution to strengthen rehabilitation within health systems globally
11. Contribution to Building an Evidence Based Approach to Rehabilitation	Contribution of academic PRM physicians to the training in Evidence Based Medicine and its implementation in rehabilitation clinical practice for PRM specialists and other professionals

Second, an academic PRM organization in Europe can serve as a platform to formulate and express a common policy for national organizations of academic PRM. This policy can then be communicated to European authorities and can be used by national organizations of academic PRM in their countries. Advice may include but is not limited to (*i*) the development of comprehensive rehabilitation services from the acute hospital to the community (3) and financed under the principle of universal health coverage, (*ii*) the education and training of the rehabilitation workforce, aiming for a balanced workforce across rehabilitation professions, (*iii*) the utilization of rehabilitation indicators for reporting that need to be included in health information systems across European countries.

Third, as the representative and hence legitimate voice of academic PRM in Europe, it can interact and coordinate efforts with other relevant stakeholders at the European level, including the Federation of European Academies in Medicine (FEAM), academies of other academic disciplines, organizations of rehabilitation professionals, funding agencies, and patient organizations.

Fourth, as the legitimate voice of academic PRM at the European level, it can support national organizations of academic PRM in their interaction with stakeholders at the national level, including university presidents, deans of medical faculties, directors of academic hospitals and research institutes, administrators of funding agencies, and governmental policymakers.

Fifth, it can foster the development of a common understanding of rehabilitation across medical specialities and health professions, of which rehabilitation is an important part (4–6), and coordinate efforts to advocate for rehabilitation at the European level.

Sixth, it can enhance the visibility of academic PRM, attracting talented students and professionals and developing more opportunities for future generations.

Seventh, it can strengthen the definition, utilization, and understanding of the term PRM for both the academic discipline and the medical specialty across European countries where there still is some variation (1).

Eighth, this organization can also work together with other similar organizations outside Europe and may represent Europe in global efforts.

## WHY DO WE NEED A REPRESENTATIVE ORGANIZATION OF ACADEMIC PRM IN EUROPE NOW?

There are important reasons why we need to act now and develop an academic PRM organization in Europe.

From a health systems perspective, we need to build on the momentum created by the 2023 WHO resolution on “strengthening rehabilitation in health systems” (7) that resulted from the World Health Organization’s (WHO) “Rehabilitation 2030: a call for action” (8) launched in 2017. The importance of this resolution for PRM has been summarized in a recent paper (9). Table II shows a synthesis of the World Health Assembly (WHA) requests relevant to academic PRM. The WHO is requested to report to the WHA in May 2026, 2028, and 2030 on the evolution of all WHA requests. These reports will be milestones for the years to come and request immediate action. There is a unique opportunity for an academic PRM organization in Europe to contribute to the realization of the WHA resolution as WHO calls for all relevant stakeholders to act now.

**Table II:** A synthesis of the WHA urgencies, invitations, and requests (7)

The World Health Assembly		
urges member states	invites relevant stakeholders	requests the WHO Director General
to raise awareness, build national commitment … and strengthen planning for rehabilitation including its integration within national health plans and policies…	*to support Member States as appropriate, in their national efforts to implement the actions in Rehabilitation 2030: A Call for Action, and to strengthen advocacy for rehabilitation, as well as support and contribute to the WHO hosted World Rehabilitation Alliance, a multi-stakeholder initiative to advocate for health system strengthening for rehabilitation*	*to develop … and to publish, before the end of 2026, a WHO baseline report with information on the capacity of Member States to respond to existing and foreseeable rehabilitation needs*
to incorporate appropriate ways to strengthen financing mechanisms for rehabilitation services … incorporating rehabilitation into packages of essential care …	*to harness and invest in research and innovation in relation to rehabilitation, inclusive of available, affordable, and usable assistive technology, including the development of new technologies, and support Member States, as appropriate, in collecting health policy and system research to ensure future evidence-based rehabilitation policy and practice*	to develop feasible global health system rehabilitation targets and indicators of effective coverage of rehabilitation services for 2030 …
to expand rehabilitation to all levels of health …		*to develop and continuously support the implementation of technical guidance …*
*to ensure the integrated and coordinated provision of high-quality, affordable, accessible, gender-sensitive, appropriate and evidence-based interventions for rehabilitation along the continuum of care…*		to ensure that there are appropriate resources at the WHO’s institutional capacity … to support Member States … and to facilitate international collaboration in this regard
*to develop strong multidisciplinary rehabilitation skills … and strengthen capacity for … workforce*		to support Member States to systematically integrate rehabilitation and assistive technology into their emergency preparedness and response …
*to enhance health information systems to collect information relevant to rehabilitation, including … functioning, utilizing … ICF…*		*to report on progress in the implementation of this resolution to the Health Assembly in 2026, 2028, and 2030*
*to promote high-quality rehabilitation research, including health policy and systems research*		
to ensure timely integration of rehabilitation in emergency preparedness and response …		
*to urge public and private stakeholders to stimulate investment in … research and innovation …*		

All items relevant to academic PRM are in italics.

An organization of academic PRM in Europe is uniquely positioned to advocate for education on rehabilitation (*i*) of all physicians – to paraphrase the motto “nothing about us without us”, we could say “nothing about health systems without physicians”; (*ii*) of all the health workforce; and (*iii*) on functioning as the third health indicator complementing morbidity and mortality (10, 11). Functioning captures what matters to our patients and has always been at the core of PRM. It should now become the common metric for health for all medical specialties and health professions as well as the health system (12, 13). PRM is the speciality fully equipped to teach the third health indicator and create the background for future physicians, irrespective of their speciality, to build up beyond mortality and morbidity indicators.

From a PRM perspective, we need to grasp and build on the momentum created by:

1. The initiation of the ISPRM Academic Forum in 2024 committed to supporting the strengthening of academic capacity worldwide.
2. The encouraging experience of countries such as the Netherlands (2), France, and Italy, where PRM is now represented by chairs and departments across medical faculties.
3. The launch in 2024 of a Cochrane thematic group adding functioning and disability to the previous focus of Cochrane Rehabilitation, the global body for research and methodological evidence (14) initiated in 2016.
4. The development of international research initiatives, including PREPARE (15–17) and the International Spinal Cord Survey (InSCI) (18), which demonstrate how PRM academics now cooperate internationally and work as teams involving clinician scientists and rehabilitation scientists.

### Towards a representative organization of academic PRM in Europe

Some of the key considerations towards the creation of a representative organization of academic PRM include (*i*) the identification of core activities, (*ii*) its relationship with other national, European, and international organizations of academic PRM, (*iii*) its principles of governance aiming for representativity and inclusion as prerequisites to serve as the legitimate voice of academic PRM in Europe.

### Core activities

Table I gives a list of possible activities. For some activities, e.g., the “training and continuous medical education for PRM specialists”, for which the UEMS PRM Board is primarily responsible, an organization of academic PRM in Europe is expected to contribute to these, in close collaboration with the existing European organizations.

### Relationships with other national and international organizations of academic PRM and academic organizations of other academic disciplines

Table III indicates the missions of ISPRM’s Academic Forum and exemplary national organizations of academic PRM. An organization of academic PRM in Europe can fill the gap in the emerging architecture of academic PRM and collaborate with such organizations at the national and international level. An organization of academic PRM in Europe may also cooperate with other national academic organizations, including the Association of Academic Physiatry (AAP) in the United States of America as well as academic organizations of other, related academic disciplines.

**Table III:** Missions of ISPRM’s Academic Forum and examples of national organizations of academic PRM

Country	Organization’s name	Mission statement
World	ISPRM Academic Forum	The vision of the ISPRM Academic Forum is for every medical school in the world to include a discipline related to rehabilitation and functioning. The main goals are: 1. To raise awareness on the importance of academy capacity building in PRM; 2. To support PRM physicians early in their professorial careers (PhD programmes); 3. to be a worldwide network for other national and regional organizations for academic PRM
France	COFEMER – Collège Français des Enseignants en Médecine Physique et de Réadaptation	The mission of COFEMER is to ensure high-quality, standardized education for rehabilitation physicians across France. It organizes national courses covering various topics in physical and rehabilitation medicine (PRM) to guarantee uniform training. This academic body is focused on maintaining the excellence of education in PRM through seminars, hands-on training, and collaboration across regions, ensuring that future specialists are well prepared to provide comprehensive, long-term care for patients with diverse rehabilitation needs. Key activities include: **Organizing specialized training** for medical students and practitioners in the field of PRM **Developing educational resources** such as portfolios and online platforms to support both practical and theoretical learning **Ensuring high-quality training** by regularly updating the curriculum to align with current scientific knowledge and medical practices
Italy	Italian College of Professors in PRM	The College includes all Italian Full Professors in PRM. The College aims to ensure the development of the discipline of PRM through the promotion and coordination of initiatives concerning all the activities of university teaching relevant to the discipline. The College aims to promote scientific and cultural updates; to develop research activities; to strengthen PRM in the Italian Health System. Additionally, to develop and coordinate teaching activities within Degree Courses, Specialization Courses, Master’s programmes, and Advanced Training, as well as within PhD programmes related to or involving PRM. The College represents the discipline before public and private institutions, both Italian, European, and international and collaborates with national and international scientific societies and organizations that share common interests and objectives
Netherlands	Informal Network	Meeting of PRM professors across the Dutch universities several times a year
USA	AAP – Association of Academic Physiatrists	The mission of the Association of Academic Physiatrists (AAP) is to empower academic physiatrists by advancing the science and practice of physical medicine and rehabilitation (PM&R), educating future leaders, and championing physiatry to transform healthcare. AAP focuses on promoting mentorship, leadership, and scholarship Additionally, AAP aims to enhance the role of rehabilitation medicine in academic institutions, foster professional connections within the field, and support individual academic development

### Governance

The guiding principles for the development of a governance model of an organization of academic PRM in Europe must be representativity and inclusion, as these are key to becoming the legitimate voice of academic PRM in Europe.

Representativity refers to 2 levels: (*i*) national organizations of academic PRM and (*ii*) individuals.

Inclusion refers to the involvement of all academics across the academic career cycle and from different perspectives including (*i*) university professors, (*ii*) aspiring academics, (*iii*) clinician scientists at or affiliated with a university department, unit, or chair, (*iv*) rehabilitation scientists at or affiliated with a university department, unit, or chair.

### Realization in concert with existing organizations of PRM

As there are currently only a few national organizations of academic PRM in Europe, the realization of an organization of academic PRM would need to include both representation of national organizations and individual membership. Also, close alignment with the development of ISPRM’s new academic forum would allow for the development of synergies from the start.

An interesting model for the creation of an organization of academic PRM in Europe is the AAP (for its mission see Table III). AAP was developed because there was the need for but no representative organization of academic PRM in the USA. Over the last decades the AAP has evolved into a representative and highly credible academic organization. In the USA, AAP is now the reference organization providing advice to universities that aim to establish an academic programme in PRM and academic departments of PRM aiming to establish a research programme.

To build on the existing strength of PRM recognized as a medical specialty across European countries, an organization of academic PRM in Europe should be developed in consultation with the 2 representative organizations of PRM in Europe, the UEMS Section and Board as well as the European Society of PRM (ESPRM). A representative organization of academic PRM in Europe may also build on the long tradition of the European Academy of Rehabilitation Medicine (EARM).

### Conclusion

We are convinced that there is a need and urgency to embark on the development of a representative organization for academic PRM in Europe that can serve as the umbrella organization of PRM academics in Europe. Not building on the momentum created by WHO’s resolution, ISPRM’s Academic Forum, exemplary developments of academic PRM in some European countries, as well as international rehabilitation research initiatives, would seem like a lost opportunity that may not come back in the foreseeable future. Only as a strong, representative and inclusive academic organization at the European level can we lay the foundation of academic PRM for the future and have the political clout to realize it.
